# Analysis of epidemiological trends in human papillomavirus infection among gynaecological outpatients in Hangzhou, China, 2011–2015

**DOI:** 10.1186/s12879-017-2498-2

**Published:** 2017-06-05

**Authors:** Lili Qian, Yu Zhang, Dawei Cui, Bin Lou, Yimin Chen, Ying Yu, Yonglin Liu, Yu Chen

**Affiliations:** 1 Department of Laboratory Medicine, the First Affiliated Hospital of Zhejiang Chinese Medical University, Hangzhou, China; 2 Department of Laboratory Medicine, Center of Clinical Laboratory, the First Affiliated Hospital, School of Medicine, Zhejiang University, No. 79, Qingchun Road, Hangzhou, 310003 China

**Keywords:** Human papillomavirus, Genotype, Flow-through hybridization, Epidemiology

## Abstract

**﻿Background:**

HPV infection is the major pathogenic factor underlying cervical cancer and precancerous lesions. The cervical HPV infection rates in gynaecological outpatients from Hangzhou, China, were studied in the period from January 2011 to December 2015.﻿

**﻿Methods:**

Exfoliated cervical cells were harvested from gynaecological outpatients in Hangzhou from January 2011 to December 2015. Twenty-one HPV subtypes were detected using flow-through hybridization. The HPV infection rates in various disease groups were compared using the Chi-square test. The infection rates of different HPV subtypes in different calendar years and in different age groups were analysed using the linear-by-linear association test and gamma value.

**Results:**

A total of 43,804 patients were recruited, of whom 9752 (22.3%) were infected with HPV. The top five among the 21 HPV subtypes detected in terms of infection rates were HPV-16, −52, −58, −53 and −18. No significant differences (linear-by-linear association test) were found in the HPV infection rates when compared over the studied years (*P >* 0.05). However, the 15–24-year-old age group showed the highest HPV infection rate, and significant differences (linear-by-linear association test) were detected among the different age groups (*P <* 0.05). The HPV infection rates exhibited an upward trend in the 15–24-year-old and >24–34-year-old groups over the past five years. There were significant differences in the HPV infection rates among the disease groups (*P* < 0.05).

**Conclusions:**

HPV-16, −52 and −58 were the major HPV infection subtypes in Hangzhou, China. The 15–24-year-old age group had a relatively high HPV infection rate with an upward trend over the past five years and thus represented a population susceptible to HPV infection.

Human papilloma virus (HPV) contains a circular double-stranded DNA genome that exhibits strict organization and host specificity. More than 200 HPV genotypes have been characterized to date, and more than 40 HPV types can infect the genital area and result in genital lesions. HPV genotypes are classified as low-risk and high-risk types based on their potential to cause cancer [1, 2].

The incidence of cervical cancer worldwide ranks second in female malignancies and is exhibiting a trend towards younger ages [3]. Methods to prevent cervical cancer have received increasing attention from researchers. Epidemiological and biological investigations have shown that HPV infection is the major pathogenic factor underlying cervical cancer and precancerous lesions [4, 5]. The genotype distribution of HPV infection exhibits regional specificity. According to statistical data from the International Agency for Research on Cancer (IARC, 2007), the top five HPV subtypes in terms of infection frequency are HPV-16, −53, −52, −18 and −39 in North America, HPV-16, −18, −31, −33 and −58 in Europe and HPV-16, −52, −58, −18 and −56 in Asia.

The latest research showed that HPV-52, −16 and −58 were the HPV subtypes with the highest infection frequencies in China [6]. Vaccines against four HPV subtypes (HPV-6, −11, −16 and −18) have been used in clinics in developed countries. In the present study, we analysed the cervical HPV infection rates in gynaecological outpatients from Hangzhou, China, over the past five years. Additionally, we performed an age-stratified analysis and an analysis by disease type. The results provide objective evidence for epidemiological studies of HPV infection and the application of HPV vaccines in the study region.

## Materials and methods

### Subjects

Clinical specimens were collected from 43,804 gynaecological outpatients at the First Affiliated Hospital of Zhejiang Chinese Medical University and the First Affiliated Hospital of the Medical School of Zhejiang University from January 2011 to December 2015. A woman was considered eligible to enter the study if she a) had current or past sexual activity, b) was not pregnant at the time of enrolment, c) had never been screened or treated for cervical cancer, d) had not undergone a total uterus or cervix resection, e) agreed to undergo an HPV test and f) agreed to participate in the present study. This study was conducted in accordance with the Declaration of Helsinki and a protocol approved by the First Affiliated Hospital of Zhejiang Chinese Medical University and the First Affiliated Hospital of the Medical School of Zhejiang University (Hangzhou, China). There were 5001 cases in 2011, 6410 cases in 2012, 7863 cases in 2013, 11,402 cases in 2014 and 13,128 cases in 2015. The recruited patients were 17–88 years old, with a median age of 44 years. In accordance with a prior overseas study, [7] we organized the subjects into the following five age groups: 15–24 years, >24–34 years,>34–44 years, >44–54 years and >54 years. Regarding the disease type, the subjects were assigned to the following five groups: cervical cancer, cervical intraepithelial neoplasia grade 1 (CIN1), CIN2, CIN3 and other diseases. The other diseases included gynaecological diseases other than cervical cancer and CIN, such as uterine fibroids, ovarian cysts, endometriosis, endometrial cancer and choriocarcinoma.

### Methods

#### HPV sample collection

The opening of the cervix was exposed using a vaginal dilator. Excess secretions at the opening of the cervix were wiped away using a cotton swab. A cervical brush was inserted into the opening of the cervix and rotated clockwise 3–5 times to acquire exfoliated cervical cells. The cells were placed into a sample tube containing cell preservation solution, stored in a refrigerator at 4 °C and analysed within 3 days of collection.

#### HPV genotype testing

Samples that tested positive for β-globin were analysed by PCR amplification of HPV DNA. HPV-positive samples were confirmed by PCR with universal L1 primer MY09/11 and GP5/6 systems. DNA from HeLa and Caski cell lines was used as positive controls, and mixtures without sample DNA were used as negative controls. HPV genotypes were determined using an HPV GenoArray Test Kit (Hybribio, Chaozhou, China), according to the manufacturer’s instructions [8, 9]. Geno-Array is an L1 consensus primer-based PCR assay that is capable of amplifying 21 HPV genotypes, including six low-risk HPV subtypes including six low-risk HPV subtypes (HPV-6, −11, −42, −43, −44 and CP8304) and 15 high-risk HPV subtypes (HPV-16, −18, −31, −33, −35,-39, −45, −51, −52, −53, −56, −58, −59, −66 and −68) (Table 1).

**Table 1:** List of Probe Sequence

HPV subtype	Probe Sequence
CP8304	5′ -gcactaatagttcagttgcag-3′
HPV-6	5′ -cataagaagataccttaggacttg-3′
HPV-11	5′ -acttagcagtaacgtctcagatgt-3′
HPV-44	5′ -tagtgctcgagacacgtatcatat-3′
HPV-42	5′ -gctatcgtcatgaactatgctaga-3′
HPV-43	5′ -actgtgtcatgacacgtatcaagt-3′
HPV-16	5′ -ctatacaagtacgtcgtcgatatg-3’
HPV-52	5′ -cagagctcgagacacgtatcaact-3’
HPV-58	5′ -agcaaatgaaacagttgcagga-3’
HPV-53	5′ -ttctatcatgacacgtatcactgg-3’
HPV-18	5′ -gtatcactaagtactcgtcgaatg-3’
HPV-39	5′ -cctaagtgtagacacgtatcagtt-3’
HPV-33	5′ -ctgatcatgaactacgtcatggat-3’
HPV-31	5′ -gacttcaatagtactagtcatcgg-3’
HPV-51	5′ -tctgaatgaaacagttgctaca-3’
HPV-68	5′ -atgtgtcatgagtatcatagc-3’
HPV-66	5′ -gcatctatagtatcagttagacg-3’
HPV-56	5′ -aatggtcatgacacgtatcaagga-3’
HPV-59	5′ -gcctaatgaaacagttgcaccc-3’
HPV-45	5′ -atgtgtcatgacacgtatcatagc-3’
HPV-35	5′ -ctagtgtcatgacacgtatcatag-3’

The test was conducted in four steps as follows: (1) HPV DNA extraction, (2) PCR amplification, (3) flow-through hybridization (a hybridized membrane coated with genotype-specific probes was placed into a hybridizer for rapid nucleic acid hybridization, and the amplification products were tested by reverse dot blotting with an enzyme label to yield a coloured reaction) and (4) result interpretation. Positive test results appeared as bluish violet dots. Samples that were positive for only one HPV subtype were defined as single HPV infections, and samples that were positive for more than one HPV subtype were defined as multiple HPV infections.

### Statistical analysis

The data were analysed using SPSS 22.0 statistical software. The positive infection rate is expressed as a percentage. For multiple infection patients, the HPV-positive rate was calculated repeatedly for each genotype. The infection rates were compared based on disease group using the Chi-square test. The linear-by-linear association test and gamma value for trends were used to evaluate changes in HPV prevalence by calendar year and by age group. *P*-values were two-sided, and statistical significance was defined as *P* < 0.05.

## Results

### HPV infection rates and trends

**Fig. 1:**
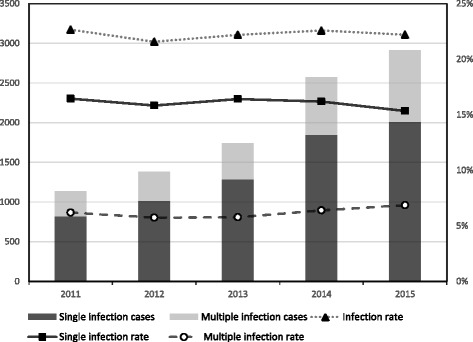
Changes in the rates of single and multiple human papillomavirus infections ingynaecological outpatients, 2011–2015

In this study, 9752 of the 43,804 women examined were infected with HPV, resulting in an infection rate of 22.3%. Specifically, the rate of single infections was 17.1% (7496/43,804), and the rate of multiple infections was 5.2% (2256/43,804). Among the 21 HPV subtypes detected, the infection rate for the low-risk subtypes was 4.5% (1987/43,804), and the infection rate for the high-risk subtypes was 20.1% (8802/43,804). Regarding the infection rate, the top five HPV subtypes were HPV-16 (4.8%, 2108/43,804), HPV-52 (4.7%, 2056/43,804), HPV-58 (3.9%, 1712/43,804), HPV-53 (2.3%, 995/43,804) and HPV-18 (1.9%, 831/43,804); the top three low-risk HPV subtypes were HPV-8304 (1.9%, 822/43,804), HPV-6 (1.3-%, 585/43,804) and HPV-11 (1.2%, 524/43,804). The HPV-positive rate in 2011 was 22.7% (1133/5001), with single infections in 16.5% (823/5001) and multiple infections in 6.2% (310/5001) of the subjects. The HPV-positive rate in 2012 was 21.6% (1383/6410), with single infections in 15.8% (1015/6410) and multiple infections in 5.7-% (368/6410) of the subjects. The HPV-positive rate in 2013 was 22.2% (1745/7863), with single infections in 16.4% (1291/7863) and multiple infections in 5.8% (454/7863) of the subjects. The HPV-positive rate in 2014 was 22.6% (2575/11,402), with single infections in 16.2% (1846/11,402) and multiple infections in 6.4% (729/11,402) of the subjects. The HPV-positive rate in 2015 was 22.2% (2916/13,128), with single infections in 15.4% (2015/13,128) and multiple infections in 6.9% (901/13,128) of the subjects (Fig. 1). No significant differences (linear-by-linear association test) were found in the HPV infection rates based on the year (*P* > 0.05) (Table 2).

**Table 2:** Infection of gynaecological outpatients with 21 subtypes of human papillomavirus (HPV) in different years

HPV subtype	Positive cases	2011 (*n* = 5001)	2012 (*n* = 6410)	2013 (*n* = 7863)	2014 (*n* = 11,402)	2015 (*n* = 13,128)	*χ* ^*2*^	*P*	gamma value
	9752	1133 (22.7%)	1383 (21.6%)	1745 (22.2%)	2575 (22.6%)	2916 (22.2%)	0.057	0.812	0.002
Low-risk									
CP8304	822	112 (2.2%)	118 (1.8%)	114 (1.5%)	197 (1.7%)	281 (2.1%)	0.228	0.633	0.028
HPV-6	585	45 (0.9%)	76 (1.2%)	140 (1.8%)	150 (1.3%)	174 (1.3%)	2.209	0.137	0.030
HPV-11	524	42 (0.8%)	83 (1.3%)	83 (1.1%)	127 (1.1%)	189 (1.4%)	7.433	0.006	-0.091
HPV-44	127	4 (0.1%)	12 (0.2%)	19 (0.2%)	43 (0.4%)	49 (0.4%)	15.253	<0.001	-0.248
HPV-42	119	13 (0.3%)	37 (0.6%)	48 (0.6%)	10 (0.1%)	11 (0.1%)	36.303	<0.001	0.410
HPV-43	35	2 (0.04%)	5 (0.08%)	4 (0.1%)	6 (0.1%)	18 (0.1%)	4.019	0.045	−0.279
High-risk									
HPV-16	2108	257 (5.1%)	311 (4.9%)	453 (5.8%)	567 (5.0%)	520 (4.0%)	15.769	<0.001	0.073
HPV-52	2056	248 (5.0%)	304 (4.7%)	328 (4.2%)	508 (4.5%)	668 (5.1%)	0.612	0.434	-0.020
HPV-58	1712	227 (4.5%)	265 (4.1%)	293 (3.7%)	454 (4.0%)	473 (3.6%)	7.423	0.006	0.046
HPV-53	995	113 (2.3%)	129 (2.0%)	148 (1.9%)	272 (2.4%)	333 (2.5%)	5.674	0.017	-0.062
HPV-18	831	91 (1.8%)	92 (1.4%)	148 (1.9%)	291 (2.4%)	209 (1.6%)	0.685	0.408	-0.007
HPV-39	765	56 (1.1%)	74 (1.2%)	78 (1.0%)	288 (2.5%)	269 (2.1%)	52.680	<0.001	-0.193
HPV-33	733	86 (1.7%)	113 (1.8%)	150 (1.9%)	187 (1.6%)	197 (1.5%)	2.691	0.101	0.049
HPV-31	611	71 (1.4%)	70 (1.1%)	141 (1.8%)	139 (1.2%)	190 (1.5%)	0.103	0.748	-0.008
HPV-51	476	11 (0.2%)	16 (0.3%)	32 (0.4%)	129 (1.1%)	288 (2.2%)	221.134	<0.001	-0.554
HPV-68	458	61 (1.2%)	75 (1.2%)	72 (0.9%)	97 (0.9%)	153 (1.2%)	0.315	0.575	0.005
HPV-66	373	44 (0.9%)	51 (0.8%)	55 (0.7%)	98 (0.9%)	125 (1.0%)	1.168	0.280	-0.049
HPV-56	270	35 (0.7%)	14 (0.2%)	43 (0.6%)	75 (0.7%)	103 (0.8%)	8.898	0.003	-0.147
HPV-59	197	11 (0.2%)	32 (0.5%)	42 (0.5%)	46 (0.4%)	66 (0.5%)	2.259	0.133	-0.068
HPV-45	122	15 (0.3%)	17 (0.3%)	33 (0.4%)	20 (0.2%)	37 (0.3%)	0.666	0.415	0.053
HPV-35	93	14 (0.3%)	16 (0.3%)	12 (0.2%)	25 (0.2%)	26 (0.2%)	0.865	0.352	0.059

### Infection of different age groups with the HPV subtypes

**Fig. 2:**
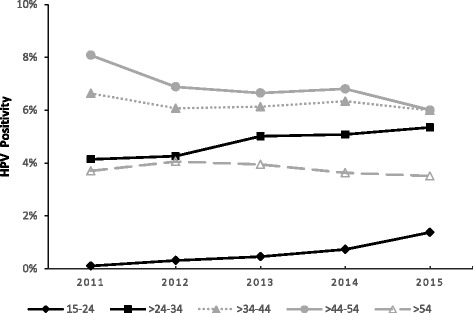
Changes in the rate of human papillomavirus infection ingynaecological outpatients in different age groups, 2011–2015

The HPV infection rates of the 15–24-, >24–34-, >34–44-, >44–54- and >54-year-old groups were 34.7% (324/933), 23.5% (2155/9159), 21.3% (2712/12,717), 20.6% (2932/14,245) and 24.1% (1629/6750), respectively. The highest infection rate appeared in the 15–24-year-old group, and significant differences (linear-by-linear association test) were found among the age groups (*P* < 0.05). Additionally, significant differences (linear-by-linear association test)were found among the age groups in the infection rates for the 11 high-risk subtypes (HPV-18, −35, −51, −52, −53, −58, −59 and −68; *P* < 0.05) and the four low-risk subtypes (CP8304, HPV-6, −11 and −42; *P* < 0.05) (Table 3). The HPV infection rates exhibited an upward trend in the 15–24- and >24–34-year-old age groups over the past five years (Fig. 2) (Table 4).

**Table 3:** Infection of gynaecological outpatients with 21 subtypes of human papillomavirus (HPV) in different age groups

HPV subtype	15–24 (*n* = 933)	>24–34 (*n* = 9159)	>34–44 (*n* = 12,717)	>44–54 (*n* = 14,245)	>54 (*n* = 6750)	*χ* ^*2*^	*P*	gamma value
	324 (34.7%)	2155 (23.5%)	2712 (21.3%)	2932 (20.6%)	1629 (24.1%)	13.606	<0.001	−0.027
Low-risk								
CP8304	35 (3.8%)	134 (1.5%)	208 (1.6%)	274 (2.0%)	173 (2.6%)	11.683	<0.001	0.100
HPV-6	57 (6.1%)	231 (2.5%)	137 (1.1%)	99 (0.7%)	64 (1.0%)	185.419	<0.001	-0.382
HPV-11	62 (6.7%)	208 (2.3%)	109 (0.9%)	86 (0.6%)	61 (1.0%)	186.174	<0.001	-0.393
HPV-42	1 (0.1%)	22 (0.2%)	33 (0.3%)	34 (0.2%)	29 (0.4%)	3.940	0.047	0.127
HPV-43	1 (0.1%)	7 (0.1%)	5 (0.04%)	11 (0.1%)	12 (0.2%)	3.832	0.050	0.243
HPV-44	3 (0.3%)	32 (0.4%)	28 (0.2%)	45 (0.3%)	19 (0.3%)	0.088	0.767	−0.014
High-risk								
HPV-16	74 (8.0%)	477 (5.2%)	547 (4.3%)	638 (4.5%)	381 (5.6%)	0.498	0.481	-0.006
HPV-18	31 (3.3%)	224 (2.5%)	217 (1.7%)	233 (1.6%)	132 (2.0%)	13.148	<0.001	-0.089
HPV-31	20 (2.1%)	127 (1.4%)	170 (1.3%)	189 (1.3%)	105 (1.6%)	0.002	0.962	0.003
HPV-33	34 (3.6%)	154 (1.7)	183 (1.4%)	221 (1.6%)	143 (2.1%)	0.025	0.874	0.015
HPV-35	4 (0.4%)	27 (0.3%)	32 (0.3%)	26 (0.2%)	6 (0.1%)	10.705	0.001	-0.248
HPV-39	30 (3.2%)	173 (1.9%)	217 (1.7%)	232 (1.6%)	121 (1.8%)	3.529	0.060	-0.045
HPV-45	7 (0.8%)	29 (0.3%)	30 (0.2%)	43 (0.3%)	16 (0.2%)	1.817	0.178	-0.072
HPV-51	39 (4.2%)	140 (1.5%)	105 (0.8%)	127 (0.9%)	73 (1.1%)	32.258	<0.001	-0.166
HPV-52	66 (7.1%)	459 (5.0%)	639 (5.0%)	603 (4.2%)	297 (4.4%)	15.420	<0.001	-0.065
HPV-53	45 (4.8%)	183 (2.0%)	244 (1.9%)	317 (2.2%)	215 (3.2%)	7.704	0.006	0.076
HPV-56	10 (1.1%)	68 (0.7%)	63 (0.5%)	81 (0.6%)	51 (0.8%)	0.286	0.593	-0.017
HPV-58	60 (6.4%)	320 (3.5%)	437 (3.4%)	531 (3.7%)	372 (5.5%)	17.682	<0.001	0.082
HPV-59	14 (1.5%)	53 (0.6%)	47 (0.4%)	52 (0.4%)	34 (0.5%)	6.424	0.011	-0.113
HPV-66	15 (1.6%)	97 (1.1%)	86 (0.7%)	94 (0.7%)	83 (1.2%)	0.254	0.615	-0.014
HPV-68	24 (2.6%)	116 (1.3%)	113 (0.9%)	127 (0.9%)	80 (1.2%)	5.572	0.018	−0.070

**Table 4:** Changes in the rate of human papillomavirus infection ingynaecological outpatients in different age groups, 2011–2015

	2011 (*n* = 5001)	2012 (*n* = 6410)	2013 (*n* = 7863)	2014 (*n* = 11,402)	2015 (*n* = 13,128)	χ^2^	*P*	*gamma value*
15–24	5 (0.1)	20 (0.3)	36 (0.5)	83 (0.7)	180 (1.4)	111.4	<0.001	0.464*
>24–34	207(4.1)	273(4.3)	394(5.0)	579(5.1)	702(5.4)	16.8	<0.001	0.064*
>34–44	332(6.6)	389(6.1)	482(6.1)	723(6.3)	786(6.0)	1.159	0.282	−0.015**
>44–54	404(8.1)	441(6.9)	523(6.7)	776(6.8)	788(6.0)	20.372	<0.001	−0.031**
>54	185(3.7)	260(4.1)	310(3.9)	414(3.6)	460(3.5)	2.587	0.108	−0.032**

Linear-by-linear Association test and Gamma Value for trend to evaluate changes in HPV prevalence by calendar year and by age group. *p < 0.05;**p > 0.05

### HPV infection in the different disease groups

**Fig. 3:**
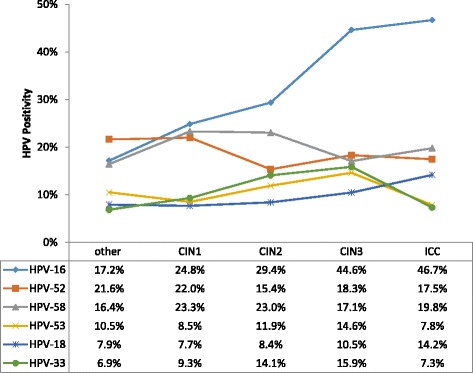
Subtype distribution of human papillomavirus (HPV) in gynaecological outpatients of different disease groups

The HPV infection rates of the different disease groups were as follows: 97.2% (889/915) in the cervical cancer group, 88.6% (287/324) in the CIN3 group, 52.7% (143/270) in the CIN2 group, 50.8% (978/1924) in the CIN1 group and 18.5% (7455/40,371) in the other diseases group. Significant differences were found in the HPV infection rates among disease groups (*P* < 0.05) and in the single and multiple infection rates among the HPV-positive patients (*P* < 0.05) (Table 5). For each disease group, the top five HPV subtypes in terms of the infection rates were as follows: HPV-16 (46.7%, 415/889), HPV-58 (19.8%, 176/889), HPV-52 (17.5%, 155/889), HPV-18 (14.2%, 126/889) and HPV-53 (7.8%, 70/889) in the cervical cancer group; HPV-16 (44.6%, 128/287), HPV-52 (18.3%, 53/287), HPV-58 (17.1%, 49/287), HPV-33 (15.9%, 46/287) and HPV-53 (14.6%, 42/287) in the CIN3 group; HPV-16 (29.4%, 42/143), HPV-58 (23.0%, 33/143), HPV-52 (15.4%, 22/143), HPV-33 (14.1%, 20/143) and HPV-53 (11.9%,17/143) in the CIN2 group; HPV-16 (24.9%, 243/978), HPV-58 (23.3%, 228/978), HPV-52 (22.0%, 215/978), HPV-33 (9.3%,91/978) and HPV-53 (8.5%,83/978) in the CIN1 group and HPV-52 (21.6%, 1611/7455), HPV-16 (17.2%, 1280/7455), HPV-58 (16.5%, 1226/7455), HPV-53 (10.5%, 783/7455) and HPV-8304 (10.0%, 745/7455) in the other disease group (Fig. 3).

**Table 5:** Single and multiple infections in human papillomavirus (HPV)-positive patients in different disease groups

	Cervical cancer^a^ (*n* = 915)	CIN3^b^ (*n* = 324)	CIN2^c^ (*n* = 270)	CIN1^d^ (*n* = 1924)	Other diseases^e^ (*n* = 40,371)	χ^2^	*P*
HPV-positive	889 (97.2)	287 (88.6)	143 (52.7)	978 (50.8)	7455 (18.5)	4557.239	<0.001
Single infections	616 (69.3)	203(70.7)	109 (76.2)	764(78.1)	5970 (80.1)	63.613	<0.001
Multiple infections	273 (30.7)	84(29.3)	34 (23.8)	214(21.9)	1485(19.9)		

Pairwise comparisons were performed using the Bonferroni method; *α* was adjusted with *P* < 0.01 considered significant. In comparing the rates of single and multiple infections, the following results were found: the difference was significant for a + d (χ^2^ = 18.820, *P* < 0.001); the difference was significant for a + e (χ^2^ = 55.598, *P* < 0.001); the difference was significant b + e (χ^2^ = 14.978, *P* < 0.001)

## Discussion

HPV is a group of DNA viruses that specifically infect human skin and the mucosal epithelium. HPV infection can cause abnormal proliferation of the skin and mucosal epithelial cells, leading to verrucous lesions and papillomas in the host tissue. Epidemiological and biological investigations have shown that HPV infection and subtype distributions are closely associated with the region, age and population [10, 11]. According to statistical data obtained from the IARC, the top five HPV subtypes worldwide in terms of infection frequency are HPV-16, −18, −58, −52 and −31. The present study was conducted in Hangzhou, which is a city located on the southeast coast of China that features a developed economy and frequent population migration. Clarifying the infection rates, genetic profiles and epidemiological patterns of HPV in Hangzhou is of great significance for the prevention and control of HPV in this region.

The present study was an epidemiological study investigating HPV infection with the largest sample size in the study area to date. Both laboratories selected for the study have achieved International Standard Organization (ISO) 15,189 accreditation. The results showed that the average HPV infection rate was 22.3% in general gynaecological outpatients from Hangzhou over the past five years. This value was close to the HPV infection rate of 22.80% in gynaecological outpatients from the same region reported by Liu et al. [12, 13] and the previously reported HPV infection rate of 26% across the country of China [6]. Our result is consistent with the HPV infection rate of 20–25% reported in general gynaecological outpatients from other countries [14]; however, it was much lower the HPV infection rate of 66.7% found in a high-risk population from this region reported by Wang et al. [15] Differences in the study populations (general gynaecological outpatients vs. a high-risk population) may be the cause of the significant difference in the HPV infection rates within the same region. Over the past three years, the rate of single infections exhibited a downward trend, whereas the rate of multiple infections showed an upward trend in the study area.

The present study showed that the infection rate of the high-risk HPV subtypes was 20.1% in Hangzhou, which was 4.43 times the infection rate found for the low-risk HPV subtypes in this region (4.5%). This ratio is higher than the previously reported ratio of high-risk/low-risk HPV infection rates in China [6]. The current results showed that the top five HPV subtypes in terms of the infection rate were HPV-16, −52, −58, −53 and −18 in Hangzhou. This result is different from the subtype distribution of HPV dominated by HPV-16, −18, −31, −33 and −58 in Europe and America and from the dominant subtype distribution of HPV-53, −52, −58, −16 and −68 in South Korea in the same Asian region [16]. Moreover, our result differed from the dominant HPV subtype distributions reported in north and southwest China [17, 18]. In the cervical cancer group, the top five HPV subtypes in terms of infection frequency were HPV-16, −58, −52, −18 and −53. HPV-16 and -18 are the most prevalent HPV subtypes worldwide and are also the most common HPV subtypes in cervical cancer patients worldwide. Our result showed that the HPV-18 infection rate ranked relatively low in Hangzhou, which was in agreement with results reported in other regions of China [19, 20].

Cervical cancer has long plagued the majority of women’s health care in China, particularly for rural women; every year, there are approximately 130,000 new cervical cancer cases [21]. The best choice for the prevention of HPV infection and treatment of cervical cancer is preventive vaccination. The US Food and Drug Administration has approved two preventive vaccine products that mainly target HPV-6, −11, −16 and −18. Clinical data have shown that the specific effect is unclear despite the protective effect of the two vaccines against infection by other HPV subtypes [22–24]. Cervarix was recently approved in China mainly against HPV-16 and -18. In the current results, the top three HPV types were HPV-16, −52 and −58 in Hangzhou, which was similar to most previous surveys in China.[6] HPV-52 and -58 were more prevalent than the vaccine type HPV-18. We expect to provide objective evidence to enhance the hypothesis that second-generation HPV prophylactic vaccines including HPV-52 and -58 may offer higher protection for women in Hangzhou and other parts of China.

Age is an important factor associated with HPV infection. One point of view is that young women have frequent sex. Reports in the literature suggest that young women are more prone to have multiple partners [25]; additionally, their immune systems are susceptible to HPV infection because they are non-sensitized. Consequently, the HPV infection rate is high in women in the 15–24-year-old group, and the rate decreases with age [26, 27]. From another perspective, women in the menopausal period have reduced immune functions, with decreased viral clearance rates and increased HPV infection rates. Therefore, there are two peaks in the age distribution of HPV infection: 15–24 year olds and women >54 years [28, 29]. In the present study, the HPV infection rates in the different age groups showed the highest HPV infection rate of 34.7% in the 15–24-year-old group, followed by an infection rate of 24.1% in the >54-year-old group. The HPV infection rate in the 15–24-year-old group showed an annual upward trend over the past five years. This result suggested that HPV infection exhibited a trend towards younger patients and that the 15–24-year-old group was the population most susceptible to HPV infection. The prevalence in the >54-year-old group should also arouse attention and suggest that HPV testing is clinically valuable for perimenopausal women in cervical cancer screening programmes.

In the present study, the HPV infection rate varied in different disease groups. The highest HPV infection rate appeared in the cervical cancer group (97.2%). This rate was close to the rate reported in the literature of 99.7% [30]. Whether multiple infections with HPV can increase or promote the development of cervical cancer is currently a controversial issue. In the present study, differences were detected in the rates of single and multiple infections among the HPV-positive patients in the different disease groups. The cervical cancer group showed the highest rate of multiple infections. This result is in agreement with previous results [18, 19]. Among the five disease groups, the top three HPV subtypes in terms of infection were HPV-16, −52 and −58. The rate of HPV-16 infection was 2.7 times the rate of HPV-52 infection (46.7%/17.5%) and 2.4 times the rate of HPV-58 infection in the cervical cancer group (46.7%/19.8%).

The present study evaluated HPV infection in gynaecological outpatients in Hangzhou, China, from 2011 to 2015. This study was an investigation of HPV infection with the largest sample size in this region to date. The results showed that HPV-16, −52 and −58 were the dominant HPV infection subtypes in Hangzhou, which provided objective evidence for the application of preventative vaccines in this region. Moreover, we found that the HPV infection rate progressively increased every year in the age groups below 34 years. HPV infection exhibited a trend towards younger patients, and the 15–24-year-old group was a population susceptible to HPV infection. These findings provide objective evidence for the selection of this age group for vaccination in the study region.

## Abbreviations

CIN: Cervical intraepithelial neoplasia
HPV: Human papillomavirus
IARC: International Agency for Research on Cancer
ISO: International Standard Organization
